# Anti-Inflammatory Effects of Red Rice Bran Extract Ameliorate Type I Interferon Production via STING Pathway

**DOI:** 10.3390/foods11111622

**Published:** 2022-05-30

**Authors:** Amnart Onsa-Ard, Rungthip Thongboontho, Narongsuk Munkong, Kanokkarn Phromnoi, Atcharaporn Ontawong, Sirinat Pengnet, Arthid Thim-Uam

**Affiliations:** 1 Division of Biochemistry, School of Medical Sciences, University of Phayao, Phayao 56000, Thailand; nard27@hotmail.com (A.O.-A.); rungchaos@hotmail.com (R.T.); kanokkarn.ph@up.ac.th (K.P.); 2 Department of Pathology, School of Medicine, University of Phayao, Phayao 56000, Thailand; jittmunkong@gmail.com; 3 Division of Physiology, School of Medical Sciences, University of Phayao, Phayao 56000, Thailand; ontawongao@gmail.com (A.O.); nan_1801@hotmail.com (S.P.)

**Keywords:** STING, red rice bran, type-I interferon, inflammation

## Abstract

Type I interferons (IFNs-I) are inflammatory cytokines that play an essential role in the pathogenesis of inflammation and autoimmune diseases. Signaling through nucleic acid sensors causes the production of IFNs-I. A stimulator of interferon genes (STING) is a DNA sensor that signals transduction, leading to the production of IFNs-I after their activation. This study aims to determine the anti-inflammatory effects of red rice bran extract (RRBE) on macrophages through the activation of STING signaling. RAW264.7 macrophage cells were stimulated with STING agonist (DMXAA) with and without RRBE. Cells and supernatant were collected. The level of mRNA expression was determined by qPCR, and inflammatory cytokine production was investigated by ELISA. The results indicate that RRBE significantly lowers the transcription of STING and interferon-stimulated genes (ISGs). Moreover, RRBE suppresses the phosphorylation of STING, leading to a decrease in the expression of *Irf3*, a transcription factor that initiates IFN-I signaling. Our results provide evidence that red rice bran extract may be a protective compound for inflammatory diseases by targeting STING signaling.

## 1. Introduction

Inflammation is the essential process for responding to pathogenic infections, cancer, autoimmune diseases, and other inflammatory diseases. Several studies suggest that the regulation of the inflammatory process involves both innate and adaptive immune systems [1,2,3]. IFNs-I are among the critical cytokines for inflammatory response and consist of IFN-α and IFN-β [4]. After secretion, IFNs-I bind to the type I IFN-α receptor (IFNAR) on target cells that signal downstream through JAK/STAT pathways to enhance the transcription of IFN-stimulated genes (ISGs), which contributes to the inflammatory responses of the body by the different mechanisms [5]. Furthermore, IFNs-I are cytokines that play a role in systemic lupus erythematosus (SLE) and rheumatoid arthritis [6]. These cytokines are mainly produced by plasmacytoid dendritic cells and macrophages [7,8]. Several investigations have indicated that patients with severe SLE express high levels of ISGs in peripheral blood mononuclear cells (PBMCs) [9,10]. In addition, several studies have revealed that activation via nucleic acid–sensor pathways induces IFNs-I production [11,12,13]. Interestingly, the stimulator of interferon genes (STING) is a cytoplasmic adaptor protein residing in the ER membrane that signals transduction to increase IFNs-I and pro-inflammatory cytokine production [14,15] through cyclic GMP–AMP synthase (cGAS)-mediated cGAMP [16,17]. However, the over-activation of STING in a vascular and pulmonary syndrome (SAVI) promotes the mRNA expression of IFN-β1 and ISGs, including CXCL10 in PBMCs [18]. However, STING-knockout mice induced with pancreatic injury showed less inflammation than STING wild-type mice [19]. Therefore, the inhibition of the STING-mediated pathway involved in inflammation using functional foods could be further investigated for therapeutic opportunities. Bioactive compounds, including flavonoids, proanthocyanidins, and anthocyanins, have been established as having anti-inflammation and antioxidant effects in several models [20,21,22,23]. Among functional food, rice (*Oryza sativa* L.) is one of the major dietary components in many countries. Several studies report that rice and its bran are an abundant source of bioactive compounds. Moreover, pigmented rice (brown, red, and purple rice) contains various bioactive compounds such as anthocyanins, proanthocyanidins [24], γ-oryzanol, vitamin E [25], tocopherols, and tocotrienols [26], which are concentrated in the bran layer. Interestingly, red rice bran showed the highest effectiveness on the antidiabetic activity among pigmented rice [24]. Interestingly, rice bran extracts the ameliorative effects of atherosclerosis in high-fat diet (HFD)-induced mice by lowering cholesterol, triglycerides, and oxidized LDL [27]. In addition, the anti-inflammatory activity of proanthocyanidin from red rice extract decreased the production of TNF-α and IL-6 by downregulating the expression of activator proteins-1 (AP-1) and nuclear factor kappa B (NF-Κb) pathways in LPS-stimulated RAW264.7 macrophage cells [28]. 

Here, we explored the functional role of red rice bran extract as an anti-inflammatory in the activation of STING in RAW264.7 macrophages, and observed this by several in vitro experiments. Furthermore, the phosphorylation of activated STING was also performed. The data from this study provide evidence that STING can be targeted using the extracts from red rice bran for the future treatment of inflammatory diseases.

## 2. Materials and Methods

### 2.1. RRBE Preparation

Red rice was obtained from a red glutinous rice variety (Ban Dok Bua, Ban Tun sub-district, Muang Phayao district, Phayao, Thailand). Red rice bran was extracted by 50% ethanol. Briefly, 1 kg of red rice was mixed in 50% ethanol solution (6000 mL) at room temperature for three days. After that, the extracts were filtered through Whatman filter paper number 1 (GE Healthcare UK Ltd., Buckinghamshire, UK) using a vacuum filtration apparatus twice. Then, the samples were concentrated in a rotary evaporator (R-100, BUCHI Labortechnik AG, Flawil, Switzerland). Finally, the extracts were lyophilized to concentrated extracts, and stored at −80 °C prior for further experiments.

### 2.2. Determination of the Total Flavonoid Content

The total flavonoids from RRBE were examined using the aluminum chloride colorimetric method. Briefly, RRBE (250 µL) was incubated with 75 µL of 5% NaNO_2_ for 6 min at RT in the dark. Then, we added 150 µL of 10% aluminum chloride (AlCl_3_) and the reaction mixture was incubated for 5 min at RT in the dark. Next, 500 µL of 1 M NaOH and 1525 µL of dH_2_O were added to the reaction mixture. The reaction mixtures were determined by a Varioskan Flash microplate reader (Thermo Fisher Scientific, Waltham, MA, USA) at 510 nm. The amount of total flavonoid content from RRBE was calculated using the catechin standard calibration plot and indicated as mg catechin/g extract.

### 2.3. Determination of the Total Phenolic Content

The total phenolic content from the RRBE was tested using the Folin–Ciocalteu method. Briefly, the RRBE (200 µL) was incubated with 1 mL of 10% Folin–Ciocalteu. Then, we added 800 µL of 7.5% Na_2_CO_3_ and the reaction mixture was incubated for 15 min at RT in the dark. Next, the absorbance signals were detected with a microplate reader (Thermo Fisher Scientific, Waltham, MA, USA) at 750 nm. The concentration of the total phenolic content from the RRBE was calculated using the gallic acid standard calibration plot and indicated as mg gallic acid/g extract.

### 2.4. Determination of the Total Proanthocyanidin Content

The total proanthocyanidin content from the RRBE was examined using a vanillin assay. Briefly, the RRBE (40 µL) was incubated with 100 µL of 1% and 100 µL of 9 M sulfuric acid (H_2_SO_4_), and the reaction mixture was measured with a Varioskan Flash microplate reader (Thermo Fisher Scientific, Waltham, MA, USA) at 490 nm. The amount of total proanthocyanidin content from the RRBE was calculated using the catechin standard calibration plot and indicated as mg catechin/g extract.

### 2.5. Cell Culture

RAW 264.7 macrophage cells (1 × 10^5^ cells/mL) were cultured in complete media containing Dulbecco’s modified Eagle’s medium (DMEM) (HyClone, Logan, UT, USA), 10% fetal bovine serum (FBS) (Gibco–Thermo Fisher Scientific, Waltham, MA, USA), 1X anti-antibiotics (Gibco–Thermo Fisher Scientific, MA, USA) and maintained at 37 °C with 5% CO_2_. RAW264.7 cells were pretreated with RRBE for 1 h followed by adding 10 μg/mL of STING agonist or DMXAA (5,6-Dimethylxanthenone-4-acetic acid) (InvivoGen, San Diego, CA, USA) for 24 h. DMSO (vehicle)-treated cells were used as the control for all subsequent in vitro experiments

### 2.6. Determination of Cell Viability by the MTS Assay

RAW 264.7 macrophage cells (1 × 10^5^ cells/mL) were cultured in complete media as described above and maintained at 37 °C with 5% CO_2_. The cytotoxicity of the RRBE in macrophages was investigated by the MTS assay following the manufacturer’s instructions (Promega, cat. G3580, Madison, WI, USA). Briefly, macrophages were stimulated with the different doses of the RRBE for 24 h. Then, cells were washed twice with media and 20 μL of MTS reagents was added into each sample in 100 μL of the culture medium. After incubation for 3 h at 37 °C with 5% CO_2_, the absorbance signals were measured at 490 nm using a Varioskan Flash microplate reader (Thermo Fisher Scientific, Waltham, MA, USA). Untreated cells were used as the control.

### 2.7. Determination of Luciferase Activity

RAW-Lucia™ ISG cells (InvivoGen, San Diego, CA, USA) were cultured in complete media supplement with 100 mg/mL of Zeocin (Invitrogen, CA, USA) and 50 mg/mL of Normocin (InvivoGen, San Diego, CA, USA) and maintained at 37 °C with a 5% CO_2_ incubator. Cells were treated with different doses of RRBE with and without DMXAA (10 µg/mL) for 24 h. The luciferase activity was monitored in the supernatant using the QUANTI-Luc luciferase reagent detector (InvivoGen, San Diego, CA, USA) per the manufacturer’s instructions.

### 2.8. Determination of Mature Phenotypes Using Flow Cytometry

RAW 264.7 cells from all experiments (1 × 10^5^ cells) were stained with I-Ab (clone: AF6-120.1; cat. 116406) antibody, and F4/80 (clone: BM8) antibody (Bio Legend, San Diego, CA, USA). Viable cells were stained with fixable Viability Dye eFluor™ 780 (Thermo Fisher Scientific, MA USA). The immune phenotype was performed using a BD^TM^ LSR-II flow cytometer (BD Biosciences, North Brunswick, NJ, USA) and analyzed by FlowJo software (version 10, USA).

### 2.9. mRNA Expression Analysis

The total RNA was extracted by the TRIzol reagent (Thermo-InvitroGen, Waltham, MA, USA), and RNA was purified by the RNeasy mini kit (QIAGEN, Germantown, MD, USA) as per the manufacturer’s instructions. The total RNA (1 µg) was used for cDNA synthesis using iScript RT Supermix (Bio-Rad, Hercules, CA, USA). The levels of mRNA were tested by real-time PCR using SsoAdvanced Universal SYBR Green Supermix (Bio-Rad, Hercules, CA, USA) using an Applied Biosystems 7500 Real-Time PCR (Applied Biosystems). The relative amounts of target mRNA were normalized by β-actin mRNA as the housekeeping gene and determined by the 2^(−ddCt)^ method. A list of primers for the PCR included:

*Cxcl10* F: 5′-CAGTGAGAATGAGGGCCATAGG-3′

R: 5′-CGGATTCAGACATCTCTGCTCA-3′

*Mx1* F: 5′-GATCCGACTTCACTTCCAGATGG-3′

R: 5′-CATCTCAGTGGTAGTCCAACCC-3′

*IRF3* F: 5′-GCTTGTGATGGTCAAGGTTGT-3′

R: 5′-AGATGTGCAAGTCCACGGTT-3′

*IRF5* F: 5′-TTTGAGATCTTCTTTTGCTTTGGA-3′

R: 5′-GTACCACCTGTACAG TAATGAGCTCTT-3′

*IRF7* F:5′-CCCAGACTGCCTGTGTAGACG-3′

R: 5′-CCAGTCTCCAAACAGCACTCG-3′

*IFN-β* F:5′-ATGAGTGGTGGTTGCAGGC-3′

R: 5′-TGACCTTTCAAATGCAGTAGATTCA-3′

*IFN-γ* F:5′-TTGCCAAGTTTGAGGTCAACAA-3′

R: 5′-TGGTGGACCACTCGGATGA-3′

*IFN-α* F: 5’-TCTGATGCAGCAGGTGGG-3’

R: 5’-AGGGCTCTCCAGACTTCTGCTCTG-3’

*Sting* F: 5′-TGCCGGACACTTGAGGAAAT-3′

R: 5′- GTTTCCGTCTGTGGGTTCTTG-3′

### 2.10. Determination of Cytokine Production

RAW 264.7 cells (1 × 10^5^ cells/mL) were cultured, then the RRBE was treated with and without DMXAA activation for 24 h. The supernatant was collected, and the concentrations of IFN-γ and IL-10 were measured by ELISA using the IFN-γ Mouse Uncoated ELISA Kit (cat. 88-7314-88) and the IL-10 Mouse Uncoated ELISA Kit (cat. 88-7105) (Thermo Fisher Scientific, Waltham, MA, USA) according to the manufacturer’s instructions. The absorbance signals were detected using a Varioskan Flash microplate reader (Thermo Fisher Scientific, Waltham, MA, USA).

### 2.11. Determination of Nitric Oxide (NO) Production

The levels of NO were tested using a Griess reagent assay. Briefly, RAW264.7 cells (1 × 10^5^ cells/mL) were cultured in complete medium and stimulated by DMXAA with and without RRBE for 24 h. The supernatant was collected and 100 µL of supernatant was incubated with 100 µL of Griess reagents at room temperature for 10 min. The absorbance signals were measured at 540 nm using a Varioskan Flash microplate reader (Thermo Fisher Scientific, Waltham, MA, USA). Untreated cells were used as the control.

### 2.12. Immunofluorescence

RAW 264.7 cells (1 × 10^5^ cells/mL) were cultured, then underwent DMXAA treatment with and without RRBE for 3 h. After that, fixed cells were performed at room temperature for 10 min by 4% formalin (Sigma-Aldrich, Darmstadt, Germany). The fixed cells were incubated with 0.2% Triton X-100 for 10 min at room temperature and blocked the unspecific binding of the antibodies with 1% BSA for 1 h. Cells were stained with STING antibody (clone: D2P2F cat: 13647, 1:200) (Cell Signaling, Danvers, MA, USA) overnight at 4 °C. Then, the secondary antibody Alexa Fluor 488 rabbit IgG (Thermo Fisher Scientific, Waltham, MA, USA) was added for 1 h at room temperature. Next, cells were stained with 1 µM DAPI (Thermo Fisher Scientific, Waltham, MA, USA) for 5 min in the dark and the fluorescence was visualized with a confocal microscope (Carl Zeiss LMS800, Oberkochen, Germany).

### 2.13. Determination of STING by Western Blot

RAW 264.7 cells (1 × 10^6^ cells) were cultured, then underwent DMXAA treatment with and without RRBE for 3 h. Cells were lysed in buffer C (final concentration at 150 mM of NaCl, 5 mM of EDTA pH 8, 1% of Triton-X100 and 10 mM of Tris-HCL pH 7.4). Protein lysates were homogenized and centrifuged at 12,000× *g*, 15 min at 4 °C. The proteins were collected in the supernatants. Then, total protein was examined with a BCA assay (Thermo Fisher Scientific, Waltham, MA, USA). Next, 15 µg of total protein was boiled in Laemmli buffer at 95 °C for 5 min. The equal proteins were separated on a 12.5% SDS-polyacrylamide gel. Then, nitrocellulose membranes were used for transferring the proteins and blocking the unspecific binding of the antibodies with 5% BSA for 1 h. The membranes were probed with STING antibody (clone: D2P2F cat: 13647, 1:2000) (Cell Signaling, Danvers, MA, USA) and incubated at 4 °C overnight. The membranes were washed with wash buffer and probed with the fluorescent secondary antibody IRDye^®^ 680RD donkey anti-rabbit IgG (1:10,000) (LI-COR, Lincoln, NE, USA) at room temperature for 1 h. Membranes were determined by protein signals using ODYSSEY CLx (LI-COR, Lincoln, NE, USA).

### 2.14. In-Gel Digestion and Identification of Protein by Mass Spectrometry

The equal protein from cell lysates was separated on a 12.5% SDS-polyacrylamide gel. Then, the protein in gel was cut into small pieces (~0.5–1 mm^3^). A small gel particle size facilitates the removal of SDS and coomassie by 25 mM NH_2_HCO_3_. Next, protein in gels was reduced and alkylated by dithiothreitol (DTT) for 30 min at 37 °C and iodoacetamide (IA) (Sigma-Aldrich, Darmstadt, Germany) for 30 min at room temperature in the dark, respectively. These samples were further quenched with DTT for 15 min at room temperature before incubating with trypsin at a ratio of 1:50 at 37 °C for 16 h. After digestion, tryptic proteins were extracted from the gels by adding 30 µL of 50% CH3CN/1% trifluoroacetic acid (TFA). Next, the peptides from the supernatant were removed and collected in a clean LoBind tube and the extracts were concentrated on speedvac. Finally, these digested proteins were resuspended in 0.1% formic acid and subjected to LC-MS/MS (Thermo Scientific, Waltham, MA, USA). The data from mass spectrometry were analyzed by Proteome Discoverer version 2.1softwere (Thermo Scientific, Waltham, MA, USA).

### 2.15. Statistical Analysis

All statistical analyses between groups were conducted using a two-tailed Mann–Whitney test, and multiple group comparisons were performed using one-way ANOVA. The standard error of mean (SEM) was used for data presentation. Statistical analyses were performed using GraphPad Prism 8.0 (GraphPad Software, San Diego, CA, USA). A *p*-value of <0.05 was considered statistically significant.

## 3. Results

### 3.1. Bioactive Contents of Rrice Bran Extract (RRBE)

The bioactive compounds from RRBE were determined using colorimetric methods. The results showed that red rice bran extract contains an essential bioactive compound, with a total phenolic content of 51.9 ± 1.73 mg of GAE/g, total flavonoid content of 22.94 ± 2.62 mg of catechin/g, and total proanthocyanidin content of 6.52 ± 0.90 mg of catechin/g as shown in Table 1. Because the bioactive contents were identified, these extracts could be observed during anti-inflammatory activity.

### 3.2. Effects of RRBE on Cytotoxicity and the Production of Inflammatory Cytokines

**Figure 1:**
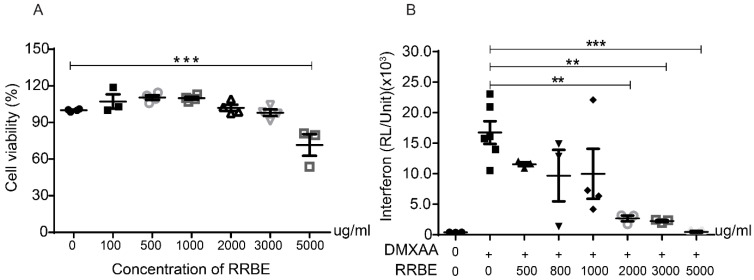
Effects of RRBE on the cytotoxicity and production of inflammatory cytokines. (**A**) The cytotoxic effects of different concentrations of RRBE were examined in comparison with the untreated group by an MTS assay (N = 3–4). (**B**) The levels of luciferase in the culture medium were determined by a luciferase detection reagent after DMXAA activation with and without RRBE for 24 h in RAW-Lucia™ ISG cells (N = 3–6). Data shown as mean ± SEM, ** *p* < 0.01, and *** *p* < 0.001.

An MTS assay was used to determine the cytotoxicity of the different doses of RRBE for 24 h on RAW 264.7 macrophage cells. The results showed that cell viabilities were not significantly affected between 100 and 3000 μg/mL of RRBE compared with the control (Figure 1A). Next, we used the different concentrations of RRBE to examine the anti-inflammatory cytokine production using STING agonist (DMXAA) activation. A previous study indicated that the appropriate concentration of DMXAA was 10 μg/mL [29]. We found that RRBE significantly decreases the levels of luciferase in the culture medium of activated RAW-Lucia™ ISG cells that were secreted under the control of interferon regulatory factor 3 (IRF3)-inducible Lucia luciferase and type I IFN production at a concentration of 2 mg/mL (Figure 1B). We used this concentration for all subsequent in vitro experiments.

### 3.3. Effects of RRBE on the Activation of RAW 264.7 Macrophage Cells

**Figure 2:**
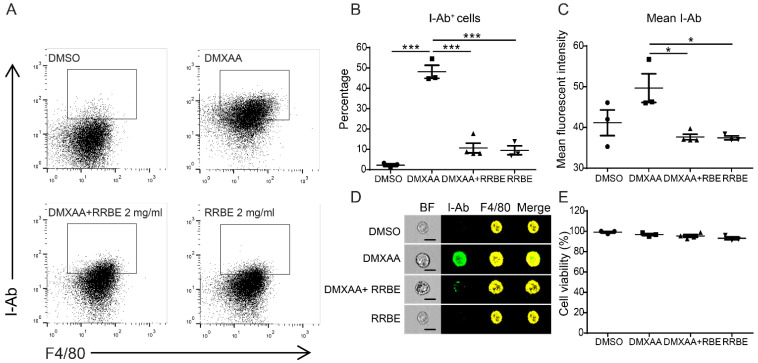
RRBE decreases the maturation of murine RAW 246.7 macrophage cells. RAW246.7 macrophages were co-stained with the macrophage surface marker (F4/80) and MHC-II molecules (I-Ab), and subsequently determined by a flow cytometer. (**A**) The representative of flow cytometry, (**B**) percentage and (**C**) mean fluorescent intensity of F4/80+ I-Ab+ cells after DMXAA stimulation with and without RRBE for 24 h (N = 3–4). (**D**) The morphology detected by imaging flow cytometry shows the representative of I-Ab (green) and F4/80 (yellow). Dead cells were excluded (N = 3) (scale bar, 20 µm). (**E**) MTS assay was used to determine cell viability (N = 3–4). Error bar shown as mean ± SEM (* *p* < 0.05 and *** *p* < 0.001).

We determined the activation of macrophage cells using flow cytometry after DMXAA activation with and without RRBE. RAW 264.7 macrophage cells were co-stained with F4/80, a surface marker identifying the mouse macrophages, and an I-Ab marker determining the activation of mouse macrophages. The treatment of macrophages with DMXAA alone showed a significant increase in the I-Ab alloantigen (MHC class II molecules), indicating fluorescent intensity, which was highly expressed on antigen-presenting cells. These surface molecules indicated macrophages activation. In comparison, the MHC-II molecules diminished in the presence of RRBE (Figure 2A–C). Moreover, we confirmed the morphology using imaging flow cytometry. The results showed that the macrophages decreased the expression of MHC-II molecules by RRBE (Figure 2D). Additionally, cell viability was tested (Figure 2E). These data suggested that RRBE diminishes the activation of RAW246.7 macrophages after the stimulation of the STING agonist by decreasing the expression of MHC-II molecules.

### 3.4. Anti-Inflammatory Effect of RRBE on RAW 264.7 Cells via STING Signaling

**Figure 3:**
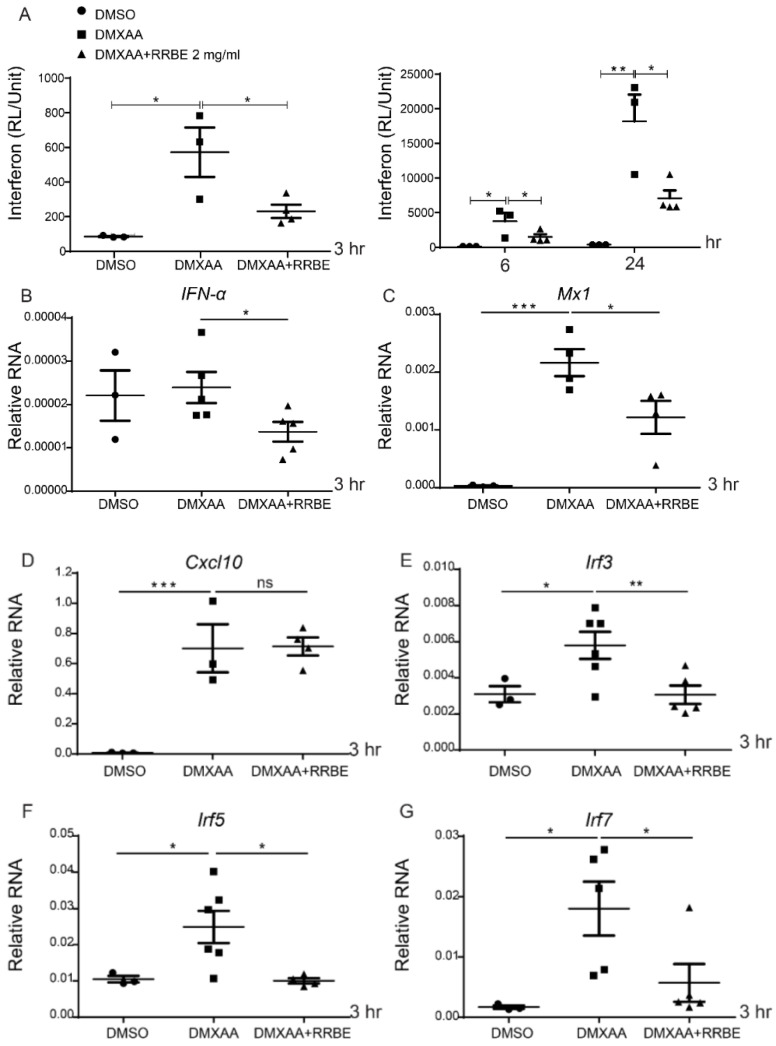
Effects of RRBE on the interferon-inducible gene expression. (**A**) The luminescence activity through STING signaling was determined after DMXAA activation with and without RRBE for 3, 6 and 24 h (N = 3–5). The relative mRNA expressions (normalized by actin) of (**B**) *Ifn-α*, (**C**) *Mx1*, (**D***) Cxcl10*, (**E**) *Irf3*, (**F**) *Irf5* and (**G**) *Irf7* after DMXAA stimulation with and without RRBE for 3 h (N = 3–4) are shown. Error bars indicate SEM, * *p* < 0.05, ** *p* < 0.01, and *** *p* < 0.001.

**Figure 4:**
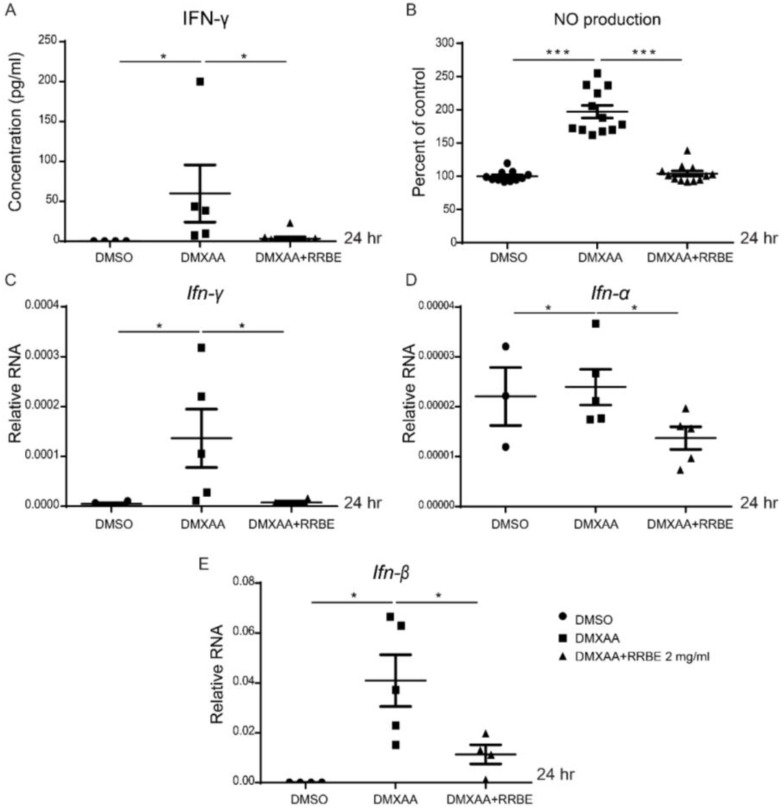
Effects of RRBE on the production of IFN-γ and nitric oxide. (**A**) Supernatants were collected, and we determined the concentration of IFN-γ after DMXAA with and without RRBE for 24 h by ELISA (N = 4–7). (**B**) Nitric oxide (NO) production was determined by the Griess assay (N = 7 to 10 per group). The mRNA expression (normalized by actin) of (**C**) *Ifn-γ*, (**D***) Ifn-α* and (**E**) *Ifn-β* are shown. Data are shown as mean ± SEM, * *p* < 0.05, and *** *p* < 0.001.

Next, the anti-inflammatory effects of RRBE via the STING pathway were investigated. The cells and supernatant were collected after the activation of DMXAA with and without RRBE. DMXAA induced the production of luciferase activity that showed the inflammatory process through type I interferon signaling. However, this induction was significantly decreased by the addition of RRBE at 3, 6, and 24 h (Figure 3A). Moreover, the mRNA levels of interferon-inducible genes (Ifn-α, Irf3, Irf5, Irf7, and Mx1) at 3 h were upregulated after DMXAA stimulation and downregulated by RRBE treatment (Figure 3B–G). Nevertheless, Cxcl10 did not alter DMXAA with RRBE treatment (Figure 3D). In addition, activation through the STING pathway enhances IFNs-I. Then, these cytokines initiate IFN-γ secretion [30], which increases the expression of iNOS and leads to nitric oxide (NO) production in activated macrophages [31]. Our study at 24 h showed that the secretion of IFN-γ and nitric oxide (NO) from DMXAA activation in the culture medium significantly increased (Figure 4A,B), while the mRNA levels of Ifn-γ, Ifn-α, and Ifn-β were downregulated in the presence of RRBE (Figure 4C–E). This finding suggested that RRBE decreased inflammatory responses through the STING-dependent pathway.

### 3.5. Effect of RRBE in the Activated Macrophages Decreases the Phosphorylation of STING

**Figure 5:**
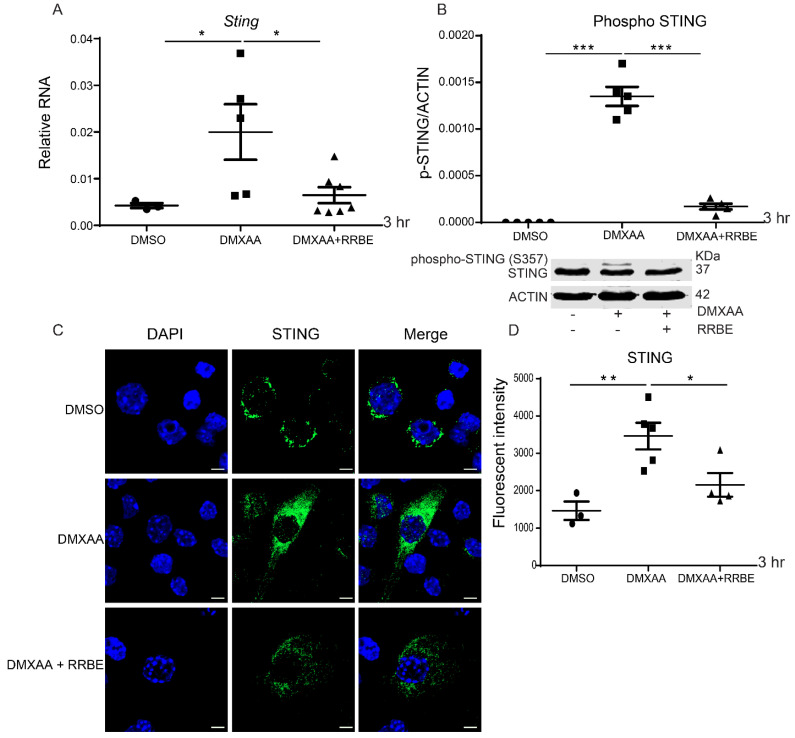
Effects of RRBE on STING activation. (**A**) The mRNA expressions of Sting are shown (N = 3–4). (**B**) The cell lysates were extracted, and Western blotting was used to analyze STING and phospho–STING protein levels after DMXAA activation with and without RRBE for 3 h (N = 3–4). (**C**) Confocal microscope of the activated cells staining RAW 246.7 cells shows STING (green) and DAPI (blue) (scale bar, 10 µm). Representations of three experiments and (**D**) the fluorescent intensity of STING expressions are shown. Data shown as mean ± SEM (* *p* < 0.05, ** *p* < 0.01 and *** *p* < 0.001).

To better understand the effect of RRBE on the STING-mediated pathway, our previous data found that STING plays a function role after being activated for 3 h [29]. Therefore, we performed the mRNA expression of Sting by real-time PCR in RAW 264.7 cells after treatment by DMXAA with and without RRBE at this time point. The expression of Sting was upregulated in the DMXAA activation compared with the presence of RRBE (Figure 5A). Additionally, we demonstrated the Western blot analysis. We found that RRBE treatment decreased the upper band of STING. This upper band, identified by mass spectrometry (LC-MS/MS)), was the phosphorylation of STING at Ser357 (data not shown) (Figure 5B). Next, we confirmed the activation of STING by confocal microscopy. These results found a higher expression of STING in DMXAA stimulation and a significant reduction in the presence of RRBE (Figure 5C). Moreover, the fluorescence intensity of the activated STING showed a significant increase in the presence of DMXAA, whereas there was a decrease in the RRBE treatment (Figure 5D). Our data suggested that the activated STING diminished in the presence of RRBE.

### 3.6. RRBE Promotes the Anti-Inflammatory Production of IL-10 in Macrophages

**Figure 6:**
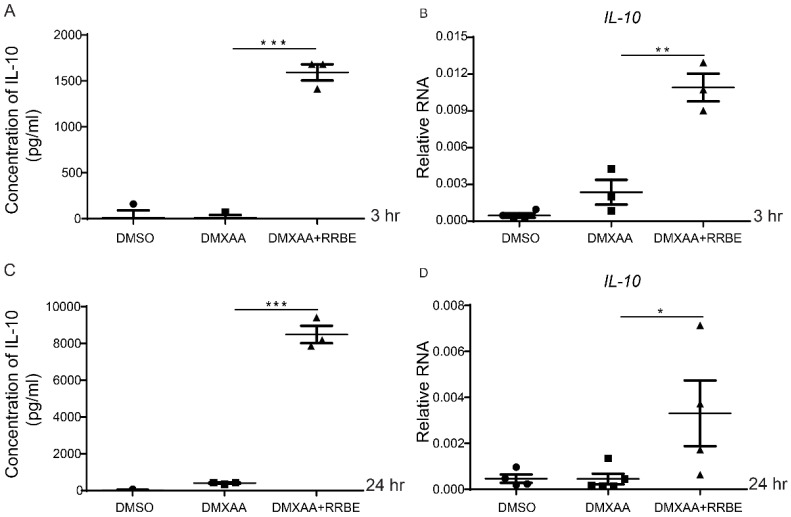
RRBE increases the production of anti-inflammation IL-10. Cells and supernatants were collected, and we analyzed the IL-10 secretion and mRNA expression by ELISA and RT-PRC after DMXAA activation with and without RRBE for (**A**,**B**) 3 h and (**C**,**D**) 24 h (N = 3–4). Data shown as mean ± SEM (* *p* < 0.05, ** *p* < 0.01 and *** *p* < 0.001).

Next, we investigated the anti-inflammatory activity of RRBE. The secretion of IL-10 from the supernatant was measured by ELISA. The results showed a significantly increased IL-10 production from the RRBE treatment with DMXAA compared to the DMXAA alone (Figure 6A,C). Furthermore, we further looked at the gene expressions and found they were significantly upregulated after treatment with RRBE (Figure 6B,D). These data suggested that RRBE enhances the mRNA expression and secretion of IL-10.

## 4. Discussion

The current study provides evidence for the possible anti-inflammatory effect of RRBE through the STING-mediated pathway in macrophage cells. The STING signaling pathway contributes to both the innate and adaptive immune systems. Several studies report the regulatory function of STING in autoimmune diseases, cancers, and inflammatory diseases [15,32,33]. Here, we detected the bioactive compounds, including total phenolic, total flavonoid, and proanthocyanidin content (Table 1). These data demonstrated that 50% ethanol extract of red rice bran contains various essential bioactive compounds similar to other colored rice extracts [34,35], showing antioxidant activity and an anti-inflammatory effect [36,37,38]. According to previous results [25,39], 40–70% ethanol extract had high total phenolic content (TPC) and exerted in vitro antioxidant and anti-inflammatory activities. Moreover, 50% ethanol fraction from red rice, which contains phenolics, proanthocyanins, and other phytochemicals, significantly increased glucose uptake in adipocytes and decreased inflammatory markers in macrophages [24,28]. In line with this, our preliminary study showed that the 50% ethanol extract of red rice bran had higher TPC compared with white rice bran and water extract (data not shown), but its TPC was not significantly different with 70% ethanol extract. Moreover, a previous study indicated that rice bran phenolic extracts decrease pro-inflammatory cytokines including monocyte chemoattractant protein 1 (MCP-1), tumor necrosis factor-α (TNF-α), interleukin-12, p70 (IL-12p70), and interferon-γ (IFN-γ) by the synergistic action of the bioactive compounds in the rice bran extract [40,41]. These results suggested that the anti-inflammatory effects observed could be due to the combination of bioactive compounds identified in red rice bran extract.

**Figure 7:**
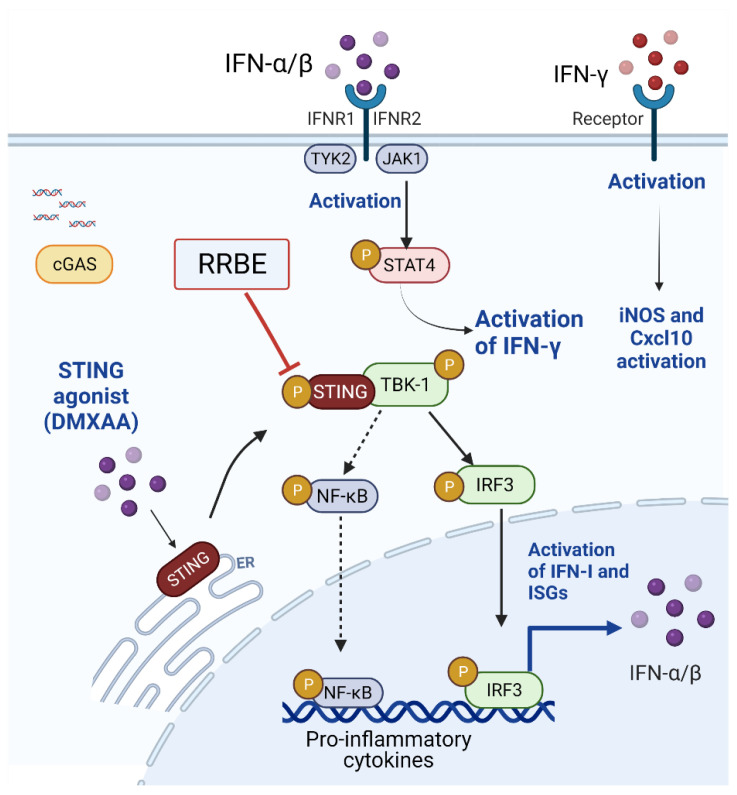
Schematic illustrations of RRBE’s effects via the STING-mediated pathway. RRBE ameliorates IFN-I production through decreasing the phosphorylation of STING. This figure was created by Biorender.com.

STING agonist (DMXAA)-stimulated RAW264.7 cells exhibit an increased expression of I-Ab (MHC-II) molecules, indicating the activation of macrophages into antigen-presenting cells [42]. The mature phenotype of activated macrophage cells was reduced in the presence of RRBE (Figure 2). Next, we observed the effects of RRBE decreasing the secretion of IFN-γ and nitric oxide in the culture medium after DMXAA-activated cells (Figure 4). Additionally, RRBE reduced the interferon-inducible genes (*Irf3*, *Irf5*, *Irf7* and *Mx1*) (*Ifn-γ*, *Ifn-β*, and *Ifn-α*) (Figure 3 and Figure 4). The increase in IFN-γ might be due to the phosphorylation of STAT4 through IFNs-I signaling (Figure 7) [7,43]. On the other hand, *Cxcl10*, which is a chemokine, did not decrease in the initial stimulation, but this gene was downregulated at 24 h after treatment (data not shown). This data may result from the decrease in IFN-γ secretion and type I IFNs (IFN-α and IFN-β) in the supernatant, which can turn on the expression of *Cxcl10* via activating the JAK/STAT pathway [44,45,46]. Moreover, the secretion of IFN-γ (after binding their receptors) can initiate the transcription of inducible nitric oxide synthase (iNOS) in activated macrophages [47] through the induced tyrosine phosphorylation of STAT1 signaling [48], and promote the production of NO (Figure 4B) [31,49,50]. These data suggested that RRBE reduced the IFN-γ and nitric oxide was STING-dependent.

This intriguing effect of RRBE via STING signaling in the inflammatory response was demonstrated. Our results showed that RRBE exhibited the anti-inflammatory effects through IFN-I signaling by lowering the mRNA expression of Sting (Figure 5A). However, we found that the total abundant protein of STING was not altered (Figure 5B). Interestingly, we detected the upper band of the STING increase in the DMXAA-treated cells and a decrease in the RRBE treatment (Figure 5B) using the total STING antibody (clone D2P2F, which recognized the STING at the C-terminal). Our previous data consistency with this result showed that this upper band, identified by mass spectrometry (LC-MS/MS), was the phosphorylation of STING at Ser357 (data not shown) [29], a homolog with S358 in human STING [51]. Subsequently, phosphorylated S357 of STING at the C-terminal (CTT, 343–379) is essential for the recruitment and phosphorylation of the downstream TANK-binding kinase 1 (TBK1) [52]. The steady state of STING resides in the ER membrane. After activation, STING trafficked to Golgi, in which the post-translational modification (PTMs) occurs for recruiting and activating TBK1. The activation, protein localization, and protein–protein interactions of STING are also regulated by several PTM processes, including ubiquitylation, SUMOylation, phosphorylation, and palmitoylation [52,53]. The data from this result assume that the Western blot from cell lysate showed another upper band of STING after the DMXAA activation could be due to the several PTMs processes. In addition, we found the RRBE treatment reduced the expression of STING by immunofluorescence staining using the STING-C antibody (Figure 5C,D). These data may result from the detection of total abundant and phosphorylated STING protein at the C-terminal. Afterward, the pSTING–pTBK1 complex activates IRF3, which is phosphorylated by TBK1. The phosphorylated IRF3 (pIRF3) dimerizes and translocates into the nucleus, initiating IFNs-I gene expression [54]. Therefore, this activation enhances IFNs-I production and other pro-inflammatory cytokines [55,56,57,58].

In this work, we found STING phosphorylation was suppressed by RRBE, which led to the lower *Irf3* expression (Figure 3E). IRF3 is the transcription factor that requires signaling through the STING-mediated pathway [54,59,60]. Our data suggested that the mRNA expression of *Irf3* in RRBE-treated macrophage cells was STING-dependent (Figure 7). These findings suggested that RRBE might affect STING function by suppressing phosphorylation, which leads to their reduced transcription downstream (Figure 7).

Previous studies revealed that proanthocyanidin-rich red rice extract decreased the pro-inflammatory cytokines, including TNF-α and IL-6, in activated macrophages by downregulating the expression of NF-Κb pathways [28]. In this work, the bioactive compounds obtained from RRBE (Table 1) showed the anti-inflammatory effects of IFNs-I production through the suppressed phosphorylation of STING. These effects lead to the downregulation of phosphorylated TBK1, which can promote the translocation of pIRF3 to the nucleus. Moreover, pTBK1 can promote NF-Κb signaling and initiate the transcription of pro-inflammatory cytokines. These findings suggested that the reduced inflammation of RRBE was STING-dependent and independent signaling.

Furthermore, we observed the level of IL-10 production in the culture medium; this is a major immunosuppressive cytokine that plays a crucial role in preventing inflammatory and autoimmune diseases [61]. We found the treatment of RRBE enhanced the production of IL-10 in DMXAA-stimulated macrophage cells (Figure 6). Enhanced IL-10 in the culture medium of RRBE-treated cells was due to higher IL-10 mRNA expression (Figure 6). A previous study showed that IFN-I through the STING-mediated pathway promotes the level of IL-10 [62,63]. In contrast, the activation of bone marrow-derived macrophages (BMDM) via cGAS-STING signaling in an allergic encephalomyelitis (EAE) mouse model showed IL-10 production without requiring IFN-I [64]. Therefore, IL-10 production can also occur through several pathways, including TLR signaling, IFN-I, and nuclear factor kB (NF-kB) [65]. Therefore, our results suggested that the increased production of IL-10 by RRBE may be STING-independent signaling. These data proposed that RRBE decreased the activated RAW264.7 macrophages’ phenotypes by reducing the production of IFNs, and this was STING-dependent.

## 5. Conclusions

This work aims to preliminarily study the anti-inflammatory effects of RRBE via the STING pathway to target a functional food component, which combines a variety of bioactive compounds. Our results indicate that the IFN-I produced via STING-mediated signaling was decreased by 2 mg/mL of RRBE treatment, and this concentration was not affected by cytotoxicity in the macrophages. We found that RRBE decreases the phosphorylation of STING, leading to a reduction in the transcription of *Irf3*, a transcription factor that initiates IFNs-I signaling. Additionally, these effects decrease the expression of interferon-inducible genes (*If-γ*, *If-β*, *If-α*, and *Mx1*). Moreover, the inflammatory cytokines, IFN-γ, and NO production diminished in the presence of RRBE. In addition, we found that the treatment of RRBE enhanced the production of immunosuppressive cytokine, IL-10. A further study via in vivo experiments to evaluate the bioactivity of RRBE for treatment involved in inflammatory disease was carried out. Therefore, blocking the STING pathway may improve inflammation, and it would probably be worthwhile to develop a therapeutic drug target using bioactive compounds from red rice bran extracts.

## Figures and Tables

**Table 1:** Bioactive compounds of RRBE.

Bioactive	Constituents
Total phenolic content (mg GAE/g)	51.9 ± 1.73
Total flavonoid content (mg Catechin/g)	22.94 ± 2.62
Proanthocyanidins (mg Catechin/g)	6.52 ± 0.90

The red rice bran was extracted by 50% ethanol and we determined the essential compounds using colorimetric methods. Data shown as means ± SD (*n* = 3).

## Data Availability

Data is contained within the article.
